# Prevalence of Newcastle Disease Antibodies in Local Chicken in Federal Capital Territory, Abuja, Nigeria

**DOI:** 10.1155/2014/796148

**Published:** 2014-10-29

**Authors:** Joseph Abraham-Oyiguh, L. K. Sulaiman, C. A. Meseko, S. Ismail, I. Suleiman, S. J. Ahmed, E. C. Onate

**Affiliations:** ^1^Department of Science Laboratory Technology, Federal Polytechnic Idah, Kogi State, Nigeria; ^2^Regional Laboratory for Avian Influenza and Other Trans-Boundary Avian Diseases, National Veterinary Research Institute (NVRI), Vom, Plateau State, Nigeria; ^3^Central Diagnostic Laboratory, National Veterinary Research Institute (NVRI), Vom, Plateau State, Nigeria

## Abstract

Newcastle disease is a contagious disease of birds and is the greatest constraint to the development of rural poultry production in Nigeria and most developing countries. The only effective means of control is vaccination which is not properly carried out in Nigeria. Therefore, this project determined the prevalence rate of Newcastle disease virus (NDV) in local chicken in the Federal Capital Territory, Abuja, Nigeria. About 5 mL of blood was collected from each of 200 chickens at the point of sale by exsanguination and sera obtained were analyzed using Haemagglutination Inhibition (HI) test to determine the prevalence of NDV. Of the 200 samples screened 34 were positive for HI antibody to NDV giving a prevalence rate of 17%. The prevalence rate obtained in this study is significant (*P* < 0.05) and indicates endemicity of the disease. There was no statistically significant (*P* > 0.05) difference in the seroprevalence of NDV antibodies among the four markets studied. Further studies are required to determine the strains circulating for appropriate preventive and control measures.

## 1. Introduction

Newcastle disease (ND) is a highly contagious viral infection of avian species especially poultry caused by Newcastle disease virus (NDV), a Paramyxovirus called avian Paramyxovirus type 1 (APMV-1). Although other host species are usually susceptible, the disease has a significant economic impact on poultry production [1]. There are about nine strains of NDV which are distinguished on the basis of pathogenicity test [2].

ND is mostly caused by velogenic strains of NDV rather than mesogenic or lentogenic strains which about 80–100% and 25% mortality, respectively, from disease [3–5]. Overall, seropositive rate of 32.5% was reported by [6] for Sokoto State, Nigeria.

Reference [4] reported a prevalence of 3.2% for NDV in clinically healthy chickens in Nsukka area, Nigeria. Reference [7] reported a higher incidence rate (68.4%) of ND during the dry season against 34.6% in the rainy season and higher rate in the young (20.7%) against 12.1% in the adult. Newcastle disease can cause great mortality in birds without any clinical signs, sometimes reaching 100 percent in unvaccinated poultry flocks and even in vaccinated poultry [4]. This disease is endemic, causing huge economic loses to farmers and hampering growth of poultry industries in Nigeria, which has an estimated poultry population of 137.6 million, with backyard poultry population constituting 84% (115.8 million) and 16% (21.7 million) of exotic poultry.

There is no means of treatment for this disease except vaccination which is not effective as outbreaks are reported yearly in vaccinated chickens. Lack of data regarding the prevalence of this disease in most parts of Nigeria has made policy formulation on controls and prevention difficult. Therefore, this research was carried out to determine the prevalence of NDV in local chicken in the Federal Capital Territory Abuja, Nigeria.

## 2. Material and Methods

### 2.1. Study Area

This study was carried out in local chicken of different sexes in Kubwa and Lugbe, Abuja-FCT, Nigeria. Abuja is located on longitude 7°, 29′ East and latitude 9°, 4′ North. The annual rainfall is high which begins from April and ends in October. The mean maximum temperature is about 27.5°C [8].

### 2.2. Sample Collection

About five milliliter (5 mL) of blood was collected into a sample bottle containing ACD from each of two hundred (200) local adult chickens by exsanguination in Kubwa and Lugbe markets in Abuja, Federal Capital Territory, Nigeria. Efforts were made to prevent discomfort to the chickens. Sera were obtained by centrifugation and transported to the Avian Viral Research Unit, National Veterinary Research Institute (NVRI), Vom, Plateau State, for laboratory analysis.

### 2.3. Haemagglutination Inhibition (HI) Test

Antibody titer for NDV was determined from each serum sample using the* OIE* HI test protocol. Briefly, 0.025 mL of PBS was dispensed into all wells of a plastic 96-well microtiter plate (v-bottomed wells) and 0.025 mL of serum was placed in the first well. 0.025 mL of the positive control serum (with known HI titer) and negative control sera were added to two respective wells of the microtiter plates. With the aid of a multichannel micro pipette, twofold dilutions of the sera were made across plate (A1–A12). The last 0.025 mL was discarded and 0.025 mL of antigen containing 4 HAU was added to all the wells except row H which serves as back titration.

Newcastle disease virus (very virulent Kudus strain) was used as antigen. Back titration was carried out; thus, 0.025 mL of antigen suspension containing 4 HAU was added into each of the first two wells of row H (4 HAU control from H1–H6), and twofold dilution was made from H2 to the H6 and the last 0.025 mL was discarded in order to obtain 4, 2, 1, 0.5, 0.25, and 0.25 HAU. 0.025 mL of PBS and albumin was added in all wells of row H and mixed by tapping gently and plates were placed at 20°C for 30 minutes. 0.025 mL of 1% washed chicken-RBC was added to each well. Mixing was done gently by tapping and the plates were placed on the bench at 20°C for 30 minutes and observed for HI.

### 2.4. Data Analysis

Geometric mean of HI antibody titer (GMT) and percentages of detectable NDV HI antibody titer were calculated. The Statistical Package for Social Sciences (SPSS) Programme (version 13) was used to compare if there was any significant difference between the geometric means of the HI antibody titer.

## 3. Result

### 3.1. Seroprevalence of NDV in Local Chicken in Kubwa and Lugbe Markets

Result showed that the seroprevalence rate of NDV antibodies for Kubwa village market was 15 positive samples (30.0%), Kubwa Monday market had 7 positive samples (14.0%), Gaso Lugbe market had 6 positive samples (12.0%), and Sabo Lugbe Market had 6 positive samples (12.0%). The mean HI titer for the respective markets includes 5.1, 4.9, 5.0, and 5.5 (Table 1).

## 4. Discussion

The present study revealed the prevalence of haemagglutination antibodies in samples from the four markets in Kubwa and Lugbe and this indicates that Newcastle disease virus infection is endemic in the area, and the markets are serving as mixing point of infected birds with susceptible ones. The sellers and buyers as well as those processing the meat are veritable vehicle of transmission of the disease. There is therefore a great threat to commercial poultry production in the Federal Capital Territory. The implication of the spread and the carrier status of the rural household chickens could be of importance considering the fact that rural chickens were reported to constitute over 90% of chicken population in Nigeria and are capable of scavenging around the environment spreading of the NDV to vaccinated and unvaccinated healthy exotic birds [9].

Haemagglutination Inhibition (HI) antibody titer between 0log_2_ and 3log_2_ is considered negative because they produce no antibody against the virus while HI antibody titer between 4log_2_ and 8log_2_ is considered positive for antibodies production against the virus based on OIE recommendation of 2000 [8, 9]. In all the four (4) markets studied an HI antibody titer of 4log_2_ and the above ones were observed and this is indicative of exposure to the virus at one time or the other and eventual production of neutralizing antibodies to protect the chicken up to the point of sale. The high HI antibody titer may be due to infection by a virulent strain of the virus such as mesogenic strains which are viruses causing clinical signs consisting of respiratory and neurological signs with low mortality and lentogenic strains which are viruses causing mild infection of the respiratory tract without visible morbidity and mortality [3]. In the US, however, the virus has been eradicated due to stringent adherence to poultry management rules and any virulent strains are of foreign origin from places where strict compliance to management regulations and good sanitary practices is lacking [10].

From the table, the overall NDV seroprevalence of 17.0% obtained was lower than the result obtained by Ibitoye et al. [11] in a retrospective study where they reported an NDV seroprevalence of 80.9% for chicken in Sokoto State. This may be due to the seasonality of NDV having high occurrence in the months of March and October [11] which coincide with onset of the rainy season and dry season, respectively. The high wind movement transfers infection from one poultry house or flock to the other [7, 9]. FCT is neighbour to Plateau State where the National Veterinary Research Institute is situated and thus could be benefiting from the surveillance services of the institute, thus stemming the tide of the disease.

The seroprevalence of NDV antibody was higher in Kubwa village market (30.0%) than in Kubwa Monday market (14.0%) and other markets studied. Reference [9] observed variation in prevalence rate of NDV from different study sites. The high rate may be due to the higher concentration of commercial poultry in Kubwa village market than the other markets studied. Higher seroprevalence rate of Newcastle disease virus antibodies was detected in both household (26.8%) and live bird markets (35.8%) and an overall seropositive rate is 32.5% by Jibril et al. [6].

Village chickens are naturally resistant and can withstand the infection without showing any clinical symptoms, thus acting as potential source of infection for commercial chicken. This means that village chickens act as host/carrier of NDV; thus, chickens raised in the backyard of farm workers could increase the threat of ND outbreaks [9, 12].

Management practices such as disease monitoring programme, appropriate prevention, and control measures should be put in place in order to prevent loss of poultry and income due to outbreaks of the disease. New birds should be quarantined and local poultry farmers should ensure that they vaccinate their flocks [9].

Most importantly, awareness programme among the farmers about the disease and routine survey to assess the degree of Newcastle disease distribution will help in planning appropriate intervention strategy.

## 5. Conclusion

Newcastle disease virus is prevalent in local chickens sold in the four markets studied in FCT which are likely to serve as host/carrier of NDV to commercial flocks. Further studies are required to determine the strains circulating in order to control them appropriately.

## Figures and Tables

**Table 1:** Seroprevalence of NDV in local chicken in Kubwa and Lugbe Markets.

Markets	Number of samples	Number of positive samples	% of positive samples	Mean HI titer
Kubwa village market	50	15	30.0%	5.1
Kubwa Monday market	50	7	14.0%	4.9
Gaso Lugbe market	50	6	12.0%	5.0
Sabo Lugbe market	50	6	12.0%	5.5

Total	200	34	17%	
